# Persistent electrical energy generation from organic diodes under constant pressure: toward organic gravity nanogenerators

**DOI:** 10.1016/j.isci.2021.102546

**Published:** 2021-05-15

**Authors:** Sooyong Lee, Hwajeong Kim, Youngkyoo Kim

**Affiliations:** 1 Organic Nanoelectronics Laboratory and KNU Institute for Nanophotonics Applications (KINPA), Department of Chemical Engineering, School of Applied Chemical Engineering, Kyungpook National University, Daegu 41566, Republic of Korea; 2 Research Institute of Environmental Science & Technology, Kyungpook National University, Daegu 41566, Republic of Korea

**Keywords:** energy resources, energy systems, energy materials

## Abstract

Here it is demonstrated that electricity can be continuously generated by pressing organic diodes with the poly(3-hexylthiophene) (P3HT) layers which are sandwiched between indium-tin oxide and aluminum (Al) electrodes. The optimized single devices with the 150-nm-thick P3HT layers are able to generate 60 μV and 45 μA by pressing, while persistent voltage (50 μV) and current (45 μA) generations are achieved by continuous pressing for 7 days. The charge generation by pressing of organic diodes is supported by the current density-voltage and capacitance measurements, while the friction of pi-orbital electrons in the P3HT chains upon pressing is proposed for the mechanism of persistent electricity generation. Organic diode modules with 14 sub-cells in series deliver ca. 0.4 V and ca. 20 μW. The present technology is expected to pave the way for next-generation energy conversion devices, organic gravity nanogenerators that enable continuous electricity generation by gravitational forces.

## Highlights

• Organic diodes with the P3HT layers can generate electricity upon compression of devices.
• The 150-nm-thick P3HT layers deliver the best voltage and current output signals.
• The optimized devices show continuous electricity generation upon pressing for 7 days.
• 0.4 V Is achieved from the organic diode modules with 14 sub-cells in series connection.

## Graphical abstract

**Figure:**
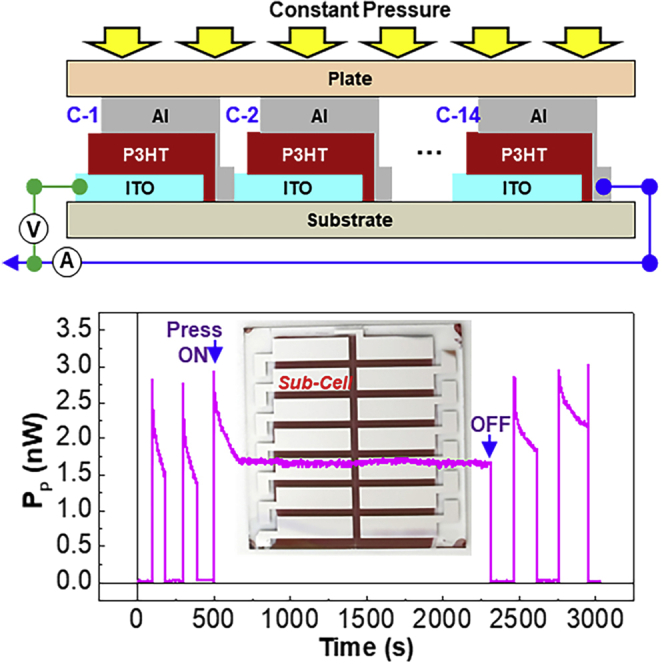

## Introduction

Generation of electrical energy has been one of the utmost important tasks in human history since the discovery of electrons and electrical bulbs (Gaurmieri, 2015, 2018). Although the power stations based on fossil and nuclear fuels have mainly contributed to the electrical energy supply, these stations are expected to be continuously minimized and will eventually disappear because of fatal environmental issues and unrecoverable disasters (Martins et al., 2019; Ramirez et al., 2019). For alternative big-scale electrical energy generation, renewable energy sources such as solar photovoltaics and wind powers are being globally installed, but there are a lot of keen concerns including inevitable environmental damages and noise issues (Wilberforce et al., 2019; Kasaei et al., 2017; Amrouche et al., 2016).

In this regard, nanogenerators based on piezoelectricity and triboelectricity have been proposed as one of the promising approaches owing to their potential for easy and eco-friendly generation of electrical energy because these devices need a mechanical pressing or rubbing component only (as an energy source) (Han et al., 2019; Patnam et al., 2020). Piezoelectricity stands for the induction of electric charges in solid materials via the rearrangement of electric dipole moments by mechanical stress (pressure) (Kim et al., 2017; Zhang et al., 2018). When piezoelectric materials such as zinc oxide and barium titanate are placed under mechanical stress, the positive and negative charge centers (dipole directions) are deformed leading to changes in electrical field for charge flow (Shin et al., 2017; Singh and Khare, 2018). However, the piezoelectric charge flow does shortly disappear after equilibrium of electric field so that applying another mechanical stress can make next charge flow (Yousry et al., 2020; Song et al., 2018). Triboelectricity, defining the electrical charge generation by friction forces, has been reported but showed a limitation similar to piezoelectricity in terms of instant charge generation (Zheng et al., 2018; Dudem et al., 2017).

Recently, it has been reported that Schottky diodes based on polymer-metal interfacial charges could generate electricity. The Schottky diodes with 1- to 2-mm-thick polypyrrole (PPy) plates, which were prepared by mechanically pressing the PPy powders and sandwiched between gold and aluminum (Al) electrodes, delivered 0.7 V and 62.4 μm/cm^2^ upon strain change but could not maintain the electricity generation after removing the strain (Shao et al., 2016). A similar approach has been reported by inserting tin oxide between PPy plates and Al electrodes, but the voltage and current signals were measured only under strain variation (note that the exact pressure information is not available) (Shao et al., 2017). Another type of energy generation based on Schottky diodes has been reported by moving the silver/graphene electrodes placed on the silicon films, with a principle which is similar to triboelectricity, even though the voltage output was only measured by the electrode movement (Lin et al., 2019).

Considering the gravitational force that is constantly applied in our daily life, it can be used as an unlimited energy source for electricity generation if a suitable energy conversion device is available (Raja et al., 2020; Park et al., 2017). However, the conventional piezoelectric and triboelectric nanogenerators cannot be actually utilized for gravitational force-based nanogenerators because of their drawbacks of instant power generation upon applying single mechanical stress. To achieve gravitational force-based nanogenerators, it is necessary to secure materials of which atoms and/or molecules under constant gravitational forces can move against the direction of gravitational force.

In this work, we demonstrate continuous generation of electricity from organic devices under constant pressure without a change in pressing intensity. The organic devices were fabricated with a geometry of conventional diode that consists of an indium-tin oxide (ITO)/poly(3-hexylthiophene) (P3HT)/Al structure. The examination of electricity generation was performed in the dark condition by employing a home-built pressing-measurement system. The evidences of charge generation were explained by current density-voltage characteristics and frequency-dependent capacitance measurements. The long-term continuous generation of electricity was examined by pressing the devices for 7 days. To prove the extension capability of the present concept, organic diode modules with 14 sub-cells were fabricated by employing a series connection architecture.

## Results and discussion

**Figure 1:**
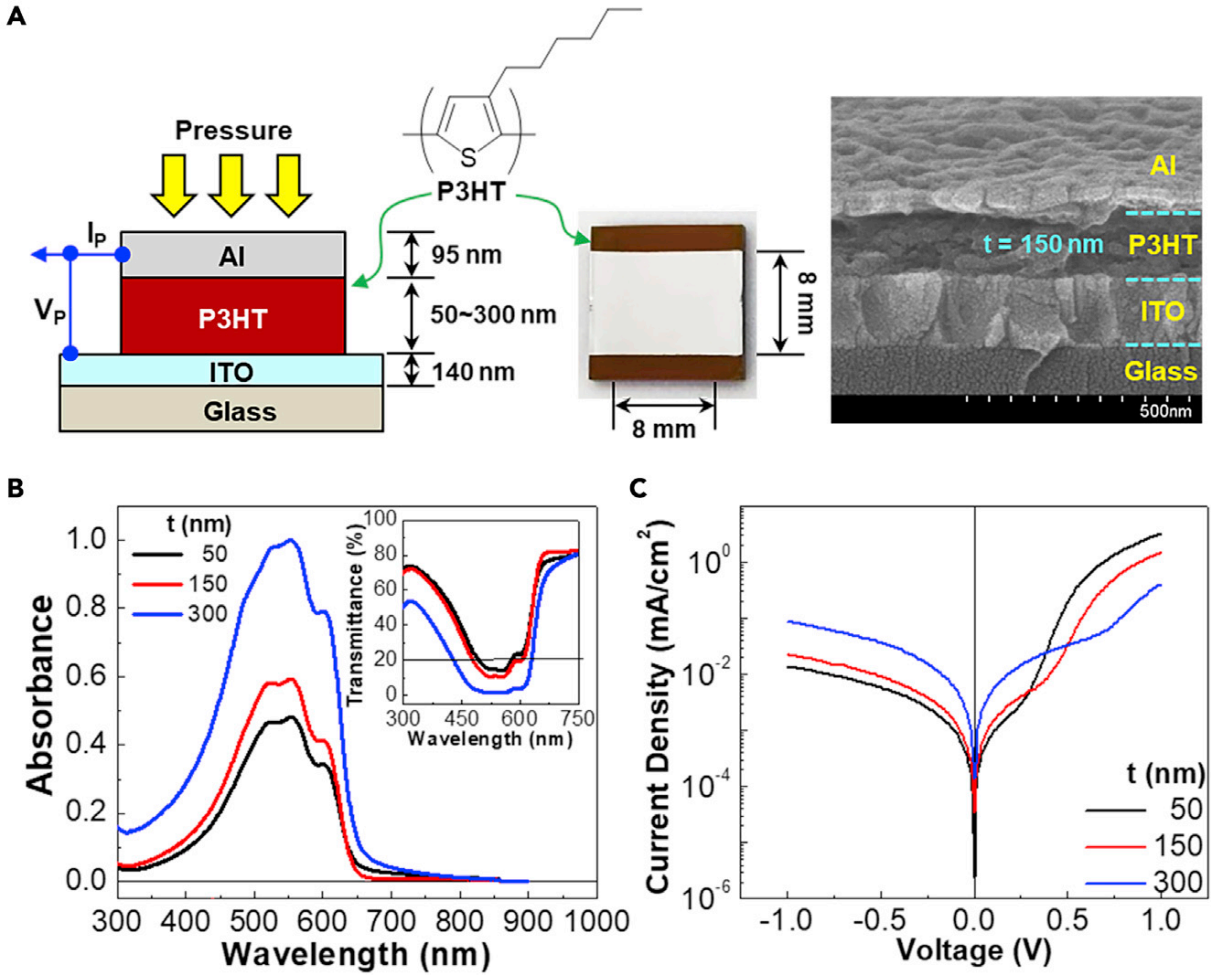
Device structure and basic characteristics for organic diodes (A) Device structure (left) for organic diodes (glass/ITO/P3HT/Al) fabricated in this work: The photograph in the middle shows the top view of device, while the cross-section part of the device with the 150-nm-thick P3HT layer is given on the SEM image. (B) Optical absorption spectra of the P3HT layers according to the P3HT thickness (inset: optical transmittance). (C) Dark current density-voltage (J-V) curves for the organic devices with the P3HT layers.

As shown in Figure 1A, organic diodes were fabricated by sandwiching the P3HT layers between the ITO and Al electrodes via typical spin-coating processes. The thickness of P3HT layers was controlled to be 50, 150, and 300 nm, while the top electrode (Al) thickness was fixed as ca. 95 nm for all devices. The optical absorption spectra showed that the absorbance of P3HT layers was increased with the thickness but the spectral shape was almost unchanged (refer Figure 1B). As plotted in the inset graph, all the P3HT layers exhibited very low optical transparency (less than 20%) at the wavelength of 500 nm. The current density-voltage (J-V) curves in Figure 1C inform that all devices show typical diode characteristics with a rectification ratio of 236.8 (50 nm), 67.7 (150 nm), and 4.71 (300 nm) at ±1.0 (refer Figure S1 for the rectification mechanism in the present organic diodes). This result reflects that the present devices were well fabricated without a physical leakage path leading to device breakdown phenomena. The well-formed P3HT layers can be supported by the cross-section part of devices (refer to the scanning electron microscope (SEM) image in Figure 1A [right]).

**Figure 2:**
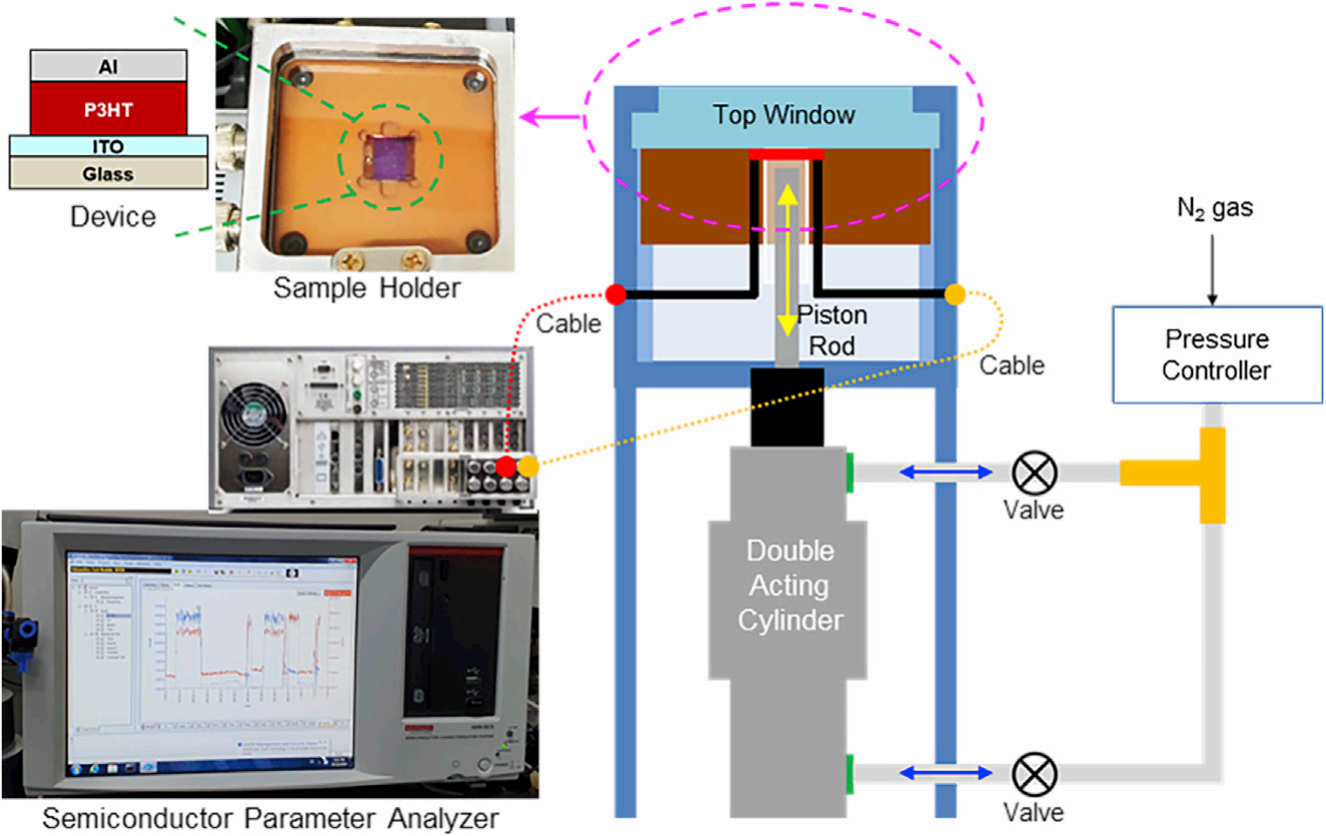
Schematic illustration for the pressure-induced electricity generation measurement system The organic devices were mounted inside a sample holder that has an inert atmosphere on the top part of the measurement system. The pressing intensity from the double-acting cylinder was controlled by the nitrogen gas via the pressure controller. The output voltage and current signals were recorded in the dark condition by a semiconducting parameter analyzer (see the left bottom part).

The devices (organic diodes) were installed inside a sample holder that is connected with a double-acting cylinder-piston assembly controlled by the nitrogen gas pressure (refer Figure 2). The pressure controller adjusts the amount of incoming nitrogen gas to pass into the double-acting cylinder, and then the piston rod moves up to the bottom part of organic diodes. The voltage and current signals, which are generated from the devices by the pressing of piston rod, are measured at the same time but individually by the semiconductor parameter analyzer (see the left bottom in Figure 2). To monitor *in situ* signals of current and voltage before and after pressing the devices, the piston rod is controlled to move up and down by programming the pressure controller. Note that all device measurements were carried out in the inert and dark condition.

**Figure 3:**
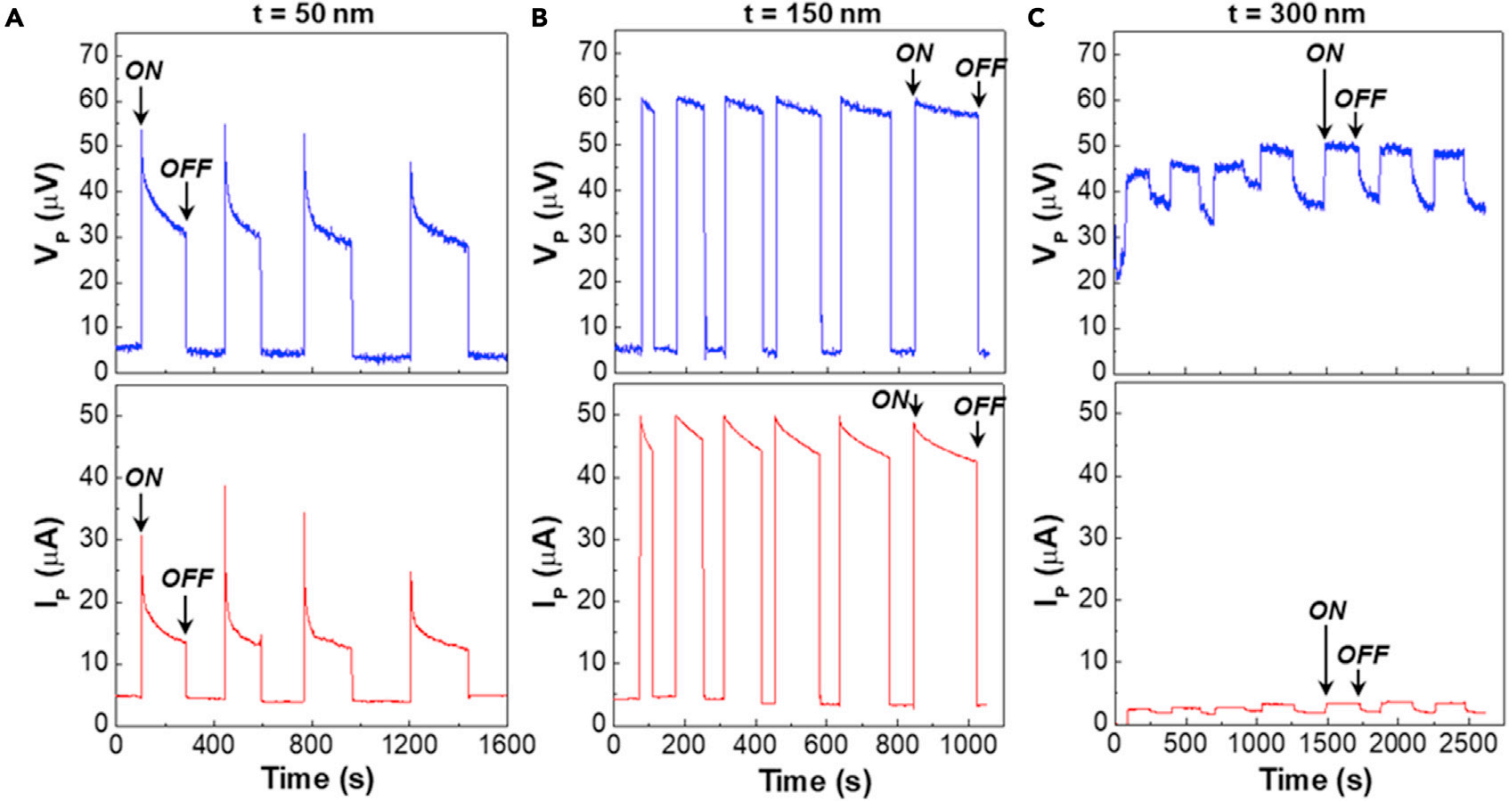
Short-term voltage and current signals generated from the organic devices with the P3HT layers by pressing (pressure = 5 kg/cm^2^) according to the P3HT thickness (t) (A) t = 50 nm, (B) t = 150 nm, (C) t = 300 nm. “ON” and “OFF” stand for pressing on (pressure = 5 kg/cm^2^) and off (pressure = 0 kg/cm^2^), respectively.

As shown in Figure 3A, the increase of voltage (V_P_) by pressing (pressure = 5 kg/cm^2^) was obviously measured for the devices with the 50-nm-thick P3HT layers. The highest V_P_ reached 55 μV (ON state) compared with 5.2 μV before pressing (note that the non-zero values before pressing can be attributed to the flow of minority charge carriers in the devices). However, the voltage was gradually decreased to 35 μV even upon pressing for 180 s and returned to the original value after removing the pressure (OFF state). The similar behavior was measured for the current (I_P_) signal. The peak current by pressing (I_P_) reached 30 μA, compared to 4.5 μA before pressing, for the devices with the 50-nm-thick P3HT layers. Interestingly, as shown in Figure 3B, the devices with the 150-nm-thick P3HT layers exhibited relatively stable V_P_ and I_P_ signals upon pressing with a smaller decay. The peak V_P_ and I_P_ values reached ca. 60 μV and 45 μA with pressing, respectively, compared to V_P_ = 5.0 μV and I_P_ = 4.3 μA without pressing. As shown in Figure S2, the stable V_P_ and I_P_ signals were also measured by pressing the devices at a pressure of 2–5 kg/cm^2^, but the highest signals were obtained at 5 kg/cm^2^. However, very poor V_P_ and I_P_ signals were measured for devices with the 300-nm-thick P3HT layers (refer Figure 3C). Here it is noted that the voltage level was still high even after removing the pressure, and the current generation was very small. The net amount of V_P_ and I_P_ was only 13 μV and 1.6 μA, respectively, for the thickest devices. These results imply that there is a transport limitation for charges generated inside devices by pressing. The optimum thickness leading to the highest V_P_ and I_P_ is 150 nm in the present study.

**Figure 4:**
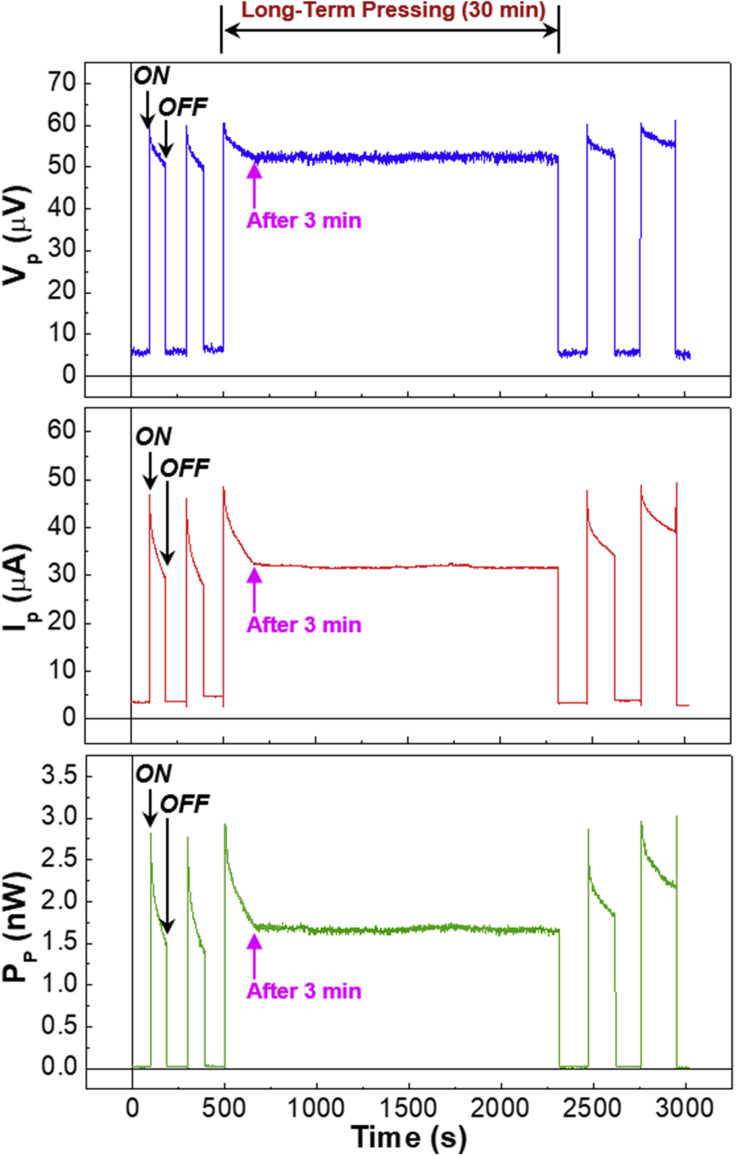
Long-term (30 min) voltage and current signals generated from the organic devices with the 150-nm-thick P3HT layers by pressing (pressure = 5 kg/cm^2^) “ON” and “OFF” stand for pressing on (pressure = 5 kg/cm^2^) and off (pressure = 0 kg/cm^2^), respectively. Note that the first and last two short-term pressing events were performed to check whether the present organic devices were still working well upon pressing. The initial decay in the device performances is marked with the pink arrows.

Based on the short-time pressing results, a longer time pressing test was carried out to investigate whether the optimized devices with the 150-nm-thick P3HT layers can continuously generate both voltage and current signals. As shown in Figure 4, the first two short-time pressing events confirm that the devices are able to generate both voltage and current signals even though there was a gradual decay upon pressing as measured in Figure 3B. Then, the third pressing was performed and then kept constant for 30 min. Very interestingly, the voltage and current signals showed an initial decay for 3 min but were stabilized for 27 min before removing the pressure. The net amount of V_P_ and I_P_ reached 50 μV and 30 μA, respectively. The resulting electrical power was 2.0 nW (see the bottom graph in Figure 4). After the 30-min pressing test, the devices could still work to generate the similar level of V_P_ and I_P_ (see the last two short-time pressing events). To further confirm much longer time operations, the devices with the 150-nm-thick P3HT layers were pressed with the same pressure (5 kg/cm^2^) for 7 days (note that the continuous data acquisition was carried out for 1 day owing to the limited storage memory in the measurement system). As shown in Figures S3 and S4 (supporting information), the output voltage and current values were almost unchanged with marginal fluctuations for the first day, which was almost similar for the other days (note that there is a noticeable gap in signals between days because the measurement system was turned off and on again for next-day measurements). Although these day-by-day drops in the voltage and current signals could be assumed actual, it is obvious that the signals became almost constant in the presence of some oscillations after the fourth day. This result indicates that the present devices can act as an energy conversion device, from constant mechanical (pressure) energy to electrical energy, and can be organic gravity nanogenerators when it comes to the continuous electrical power generation by placing a heavy material on top of the devices.

**Figure 5:**
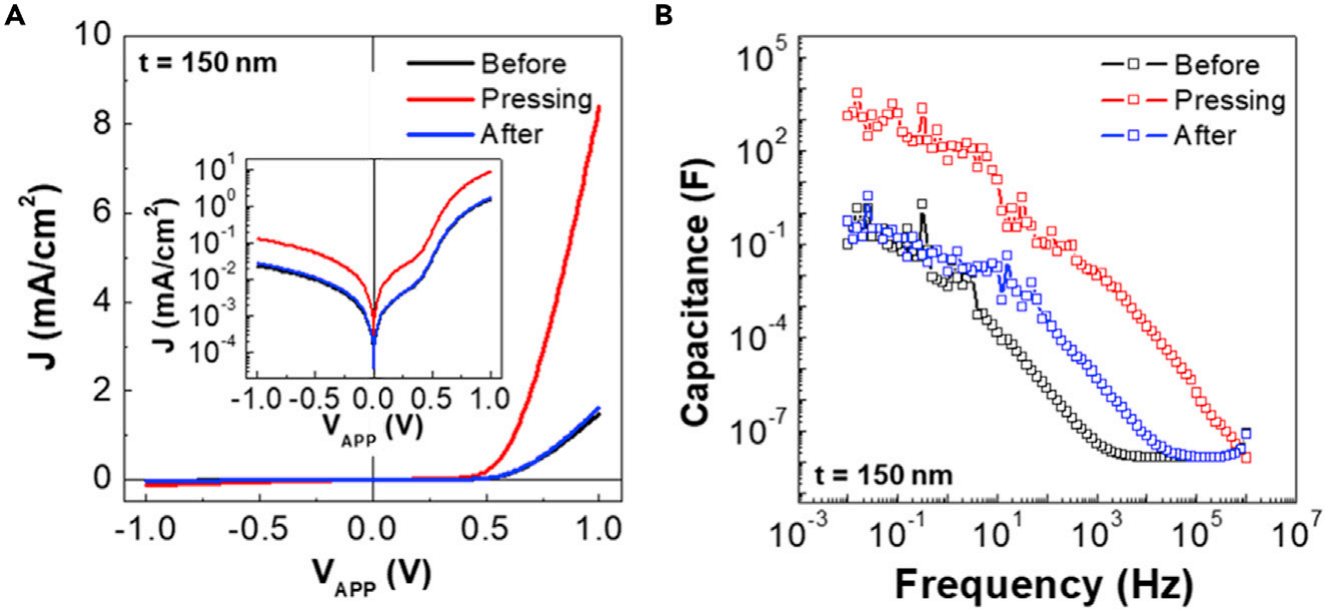
Electrical characteristics of organic diodes according to pressing conditions (A) Current density-applied voltage (J-V_APP_) curves (dark condition) before pressing (0 kg/cm^2^), upon pressing (5 kg/cm^2^), and after pressing (0 kg/cm^2^) for the organic devices with the 150-nm-thick P3HT layers (inset: semi-logarithmic scale). (B) Capacitance as a function of frequency before pressing (0 kg/cm^2^), upon pressing (5 kg/cm^2^), and after pressing (0 kg/cm^2^) for the organic devices with the 150-nm-thick P3HT layers.

To further understand, the electrical characteristics of devices were measured upon pressing. As shown in Figure 5A, the current density at forward bias (8.4 mA/cm^2^ at 1.0 V) was greatly increased by pressing compared with that (1.4 mA/cm^2^ at 1.0 V) before pressing. This huge jump in the current density (ca. 4-fold) can be ascribed to the transport of charges generated by pressing because it cannot be explained by the possible thickness reduction of the P3HT layers by pressing (only 0.01 mA/cm^2^ at 1.0 V can be increased by ca. 20 nm reduction of thickness). Particular attention is paid to the J-V curve after removing pressure because it returned to almost the same shape as original curve (before pressing). This supports that the present devices were not damaged by pressing, and the thickness was almost completely restored. To get further solid evidence, the frequency-dependent capacitance of devices was measured as shown in Figure 5B. The capacitance was greatly increased up to 1304 F at 0.01 Hz and 0.09 F at 100 Hz by pressing, compared with that before pressing (0.09 F at 0.01 Hz and 2.23 × 10^−6^ F at 100 Hz). This huge jump in capacitance can be attributed to the increased number of charges that are generated by pressing because the P3HT thickness reduction is unable to explain this phenomenon by pressing (only 5.8 × 10^−10^ F at 0.01 Hz can be increased by ca. 20 nm reduction of thickness). However, the capacitance was not perfectly restored even after removing the pressure, which can be attributed to the trapping of charges during the alternating-current-like cyclic measurement of capacitance-frequency plots (note that the J-V curves were measured with a direct current mode).

**Figure 6:**
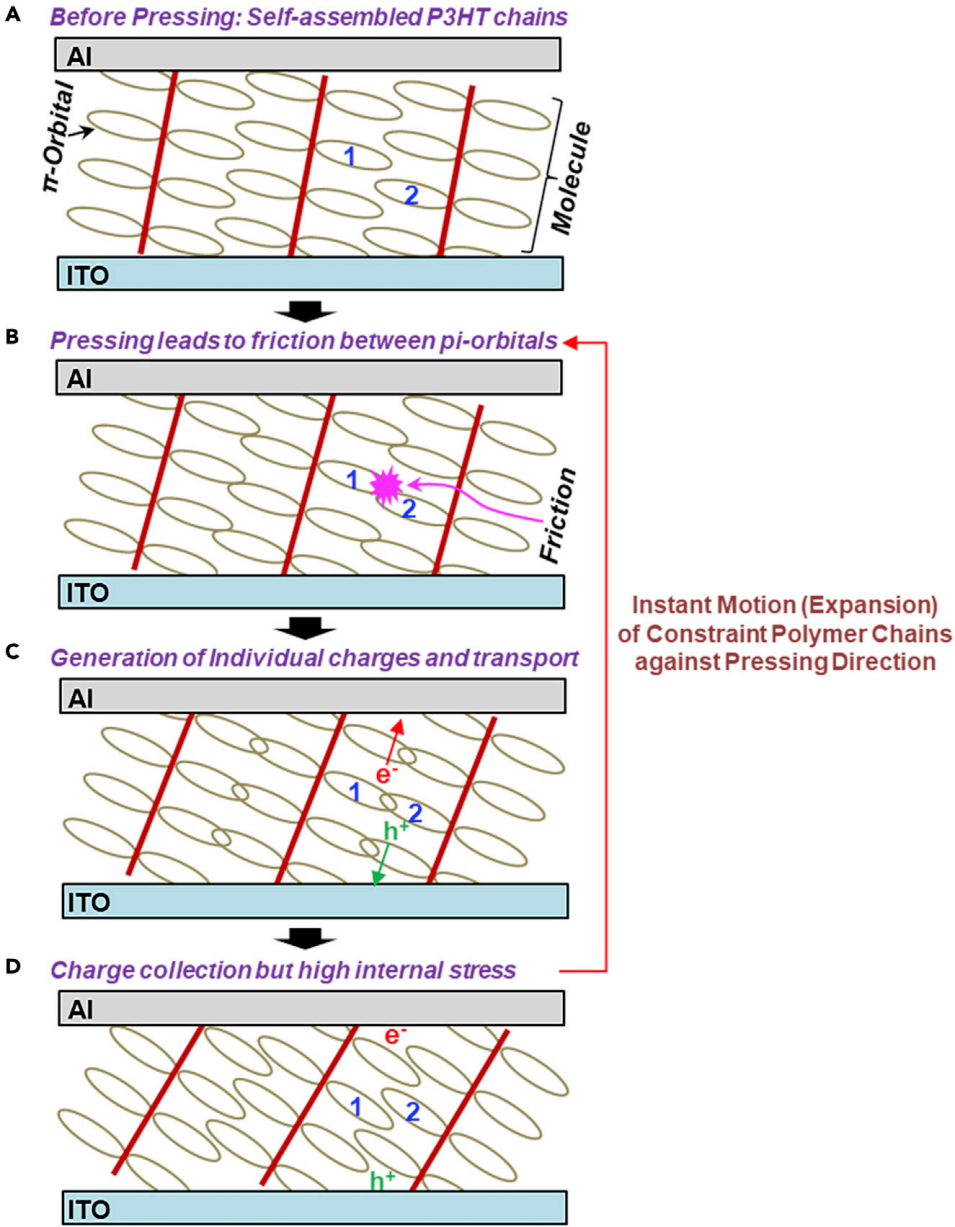
A mechanism proposed for the continuous generation of electricity upon pressing the organic devices with the P3HT layers with constant pressure (A) Before pressing: close arrangement of π-orbitals in the P3HT chains layer, (B) excited state formation by friction of π-orbitals (see “1” and “2” for example) in the P3HT chains by the pressed force, (C) charge generation from excited states and transport, and (D) charge collection by corresponding electrodes in the presence of high internal stress. Note that the constraint polymer chains under pressing try to return to the stable (original) unconstraint states by the driving force of internal stress, which enables the cycle (contraction-expansion) between (B) and (D) leading to the continuous generation of electricity.

A mechanism on the electricity generation by pressing is proposed as illustrated in Figure 6. Considering the self-assembled and well-stacked (ordered) states of P3HT chains (edge-on alignment mostly and polycrystalline), the pi-orbitals of P3HT chains may locate close to each other (Figure 6A). Upon pressing, the pi-orbitals can be further closer leading to friction among their electrons (Figure 6B). This electron friction may relocate electrons, which in turn induces mobile charges (electrons and holes) similar to triboelectricity (Figure 6C) (Huang et al., 2020). Then the mobile charges are transported to corresponding electrodes (ITO and Al) by the built-in electric field (Figure 6D). Here, the pressed P3HT chains are considered to strongly vibrate toward the opposite direction of pressing in order to restore their stable original states. Therefore, continuous processes between pressing (contraction) and restoring (expansion) actions in the P3HT films can make endless frictions in the pi-orbitals of P3HT chains leading to the persistent generation of charges upon pressing (as measured in Figure 4). Note that the gradual decay of voltage and current signals by the short-time pressing (refer Figures 3 and 4) can be explained by the initial stabilization step of P3HT chains inside as-fabricated devices. After the initial stabilization step, the P3HT chains might step into the stable contraction-expansion cycles upon constant pressure (refer to the stabilized voltage and current signals for the 30-min pressing in Figure 4). During the contraction-expansion cycles, the thickness of P3HT layers in the devices is considered to slightly change as described in Figure S5.

**Figure 7:**
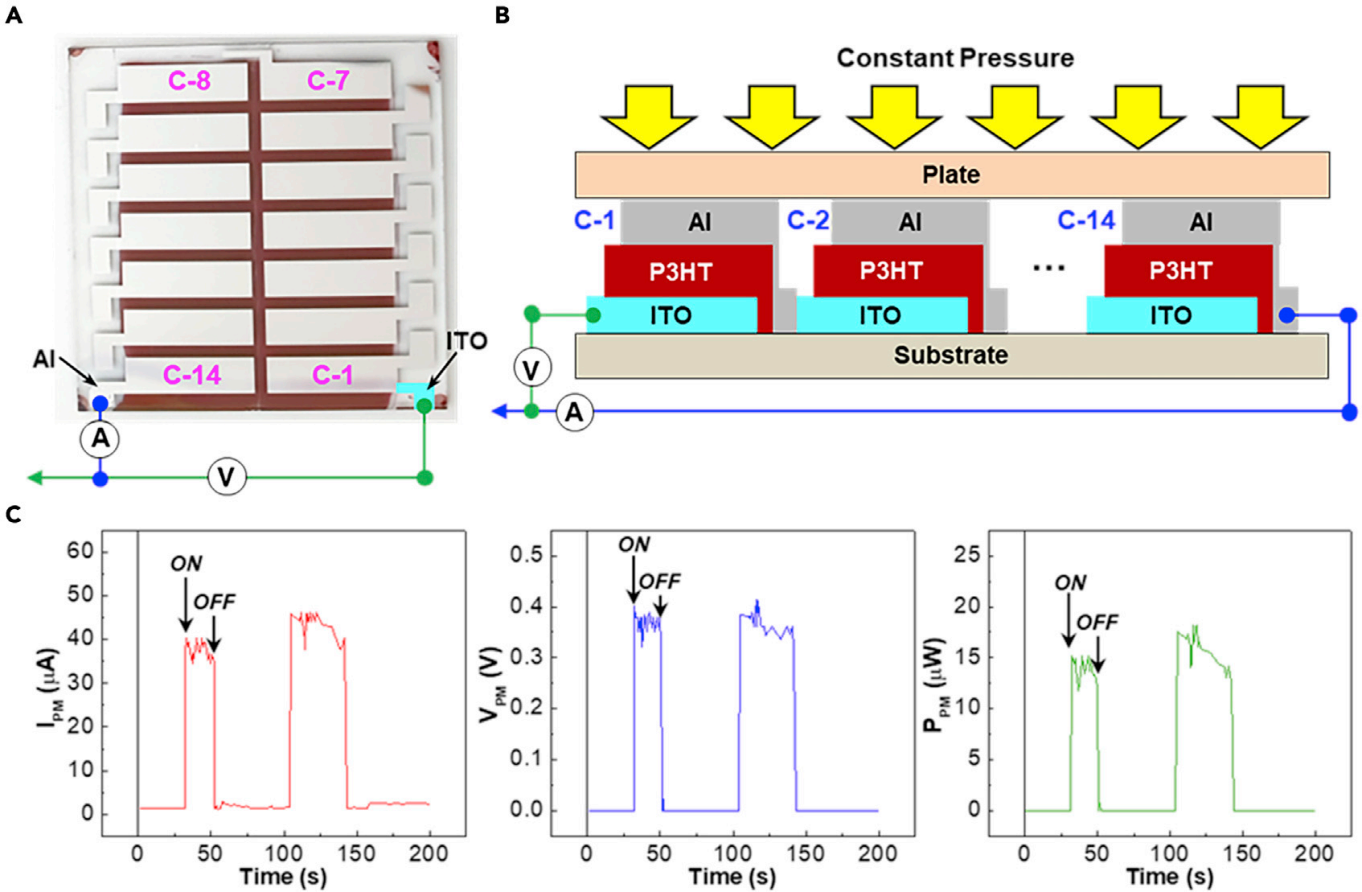
Organic diode modules with 14 single cells in series connection (A) Photograph for the organic diode module with 14 single sub-cells (C-1 ∼ C14) in series connection: Note that the signal lines were connected between the ITO electrode in C-1 and the Al electrode in C-14. (B) Schematic illustration for the organic diode module under constant pressure. (C) Current (I_PM_), voltage (V_PM_), and power (P_PM_) from the organic diode module by pressing. “ON” and “OFF” stand for pressing on (pressure = 5 kg/cm^2^) and off (pressure = 0 kg/cm^2^), respectively.

To prove and confirm that the present electricity generation is scalable, organic diode modules were fabricated by designing 14 sub-cells on single substrates (30 mm × 30 mm) as shown in Figure 7A. Note that the 150-nm-thick P3HT layers were used for the module fabrication. Because the sub-cells are connected in series, it is expected that the voltage of modules can be increased, compared to a single device, but the current will remain constant (Figure 7B). As shown in Figure 7C, the current of organic module (I_PM_) by pressing was measured ca. 35–40 μA, which is similar to that of single cell in Figures 3 and 4. However, the voltage of organic diode module (V_PM_) by pressing reached ca. 0.3–0.4 V, which is 6600-fold higher than that (ca. 60 μV) of single cell. Considering the voltage of single cell, 14 sub-cells should deliver 0.84 V theoretically. The discrepancy can be explained by the resistive loss of sub-cell connections. As a result, the present organic diode modules can generate ca. 20 μW, which is ca. 1,000 times higher than ca. 2.5 nW of single cell. This result implies that further enhancement in electricity generation can be achieved by connecting more sub-cells and stacking single modules vertically toward a 3-dimensional assembly.

### Conclusions

Pressure-induced electricity generation in organic diodes was examined by employing the P3HT layers as an active medium. All devices showed the increased voltage and current signals upon pressing. The devices with the thin P3HT layers (50 nm) exhibited a large drop in voltage and current even upon pressing, while the pressure-induced voltage and current signals were very small for the devices with the thick P3HT layers (300 nm). However, the devices with the 150-nm-thick P3HT layers delivered relatively good voltage (60 μV) and current (45 μA) signals, with a small decay by short-time pressing, upon pressing. The optimized devices with the 150-nm-thick P3HT layers exhibited very stable and continuous voltage (50 μV) and current (45 μA) signals upon continuous pressing for 30 min (marginal drop after 7 days) even though there was an initial decay for ca. 3 min. The resulting electric power was ca. 20 nW for the optimized single devices. The current density-voltage and capacitance measurements confirmed the charge generation by pressing as well as the restored device characteristics after pressing. The pressing-restoring mechanism of P3HT chains, leading to pi-orbital frictions, has been proposed to explain the present charge generation phenomenon by pressing. The organic diode modules fabricated with 14 sub-cells in series demonstrated that the voltage of modules was greatly increased up to ca. 0.4 V (compared with ca. 60 μV of single cell) leading to the electrical power of ca. 20 μW. Therefore, it is expected that the present technology of pressure-induced electricity generation from organic diodes can be a breakthrough to open organic gravity nanogenerators that enable continuous electricity generation by gravitational forces.

### Limitation of the study

Although the present work demonstrates electricity generated from organic diode devices under constant pressure, there is a limitation to provide an exact evidence for the contraction-expansion mechanism of P3HT chains (pressing-restoring of P3HT films) leading to pi-orbital frictions. This is why no instrument is available to accurately monitor the vibration of polymer chains and/or contraction-expansion of ca. 150-nm-thick polymer films. Because the bottom and top plates are placed to compress the organic diode devices, it is very difficult to directly apply a nanoscale tip in the currently available atomic force microscope or surface profilometer. However, it is expected that the continuous contraction-expansion behavior of nanoscale polymer films can be measured if an extremely precise laser interferometer or a brand-new nanoscale tip-monitoring system is constructed together with the sophisticated electrical measurement system equipped with a transparent electrode in the future. It is carefully predicted that the thickness change (displacement) in the present P3HT films of devices upon contraction-expansion cycles might fall within ca. 10% when it comes to the roughly measured piston oscillations in the double-acting cylinder system.

**Table 1:** Summary of device parameters under pressing (5 kg/cm^2^) from the organic diodes with the P3HT layers according to the P3HT thickness (t)

Parameters	Before pressing			Pressing on			Pressing off			After pressing		
	t (nm)											
	50	150	300	50	150	300	50	150	300	50	150	300
V_P_ (μV)	5.28	3.89	36.7	53.5	60.7	49.9	31.2	57.3	49.8	5.48	3.69	37.1
I_P_ (μA)	4.35	4.34	1.77	38.7	49.9	3.35	13.1	44.2	3.37	4.02	3.61	1.95
P_P_ (nW)	0.02	0.01	0.06	2.07	3.03	1.67	0.41	2.53	0.17	0.02	0.01	0.07

## STAR★Methods

### Key resources tables

**Table:** 

REAGENT or RESOURCE	SOURCE	IDENTIFIER
**Chemicals**		

P3HT	Solaris Chem Inc.	SOL4106
Chlorobenzene	Sigma-Aldrich	Cat# 270644
Aluminum	Sigma-Aldrich	Cat# 433705

**Other**		

Surface profiler	Bruker	DektakXT-E
Scanning electron microscope	Hitachi High-Technology	S-4800
UV-visible spectrometer	PerkinElmer	Lambda 750
Electrometer	Keithley	Keithley 2400
Semiconductor parameter analyzer	Keithley	Keithley 4200 SCS
Pressure controller	CHIYODA SEIKI	Model 2001

### Resource availability

#### Lead contact

Further information and requests for resources should be directed to and will be fulfilled by the lead contact, Youngkyoo Kim (ykimm@knu.ac.kr).

#### Materials availability

This study did not generate new materials.

#### Data and code availability

This study did not generate any data sets.

### Method details

#### Materials and solutions

P3HT (regioregularity = 95%, weight-average molecular weight = 5.3×10^4^ Da, polydispersity index = 1.5) was used as received from Solaris Chem Inc. (Canada). The P3HT solutions were prepared using chlorobenzene (solvent) at a solid concentration of 13.3 ∼ 33.3 mg/ml for various film thicknesses (50 ∼ 300 nm). The P3HT solutions were subjected to continuous stirring with a magnetic bar prior to spin-coating.

#### Film and device fabrication

For the fabrication of organic devices and modules, the pre-patterned indium tin oxide (ITO)-coated glass substrates were immersed into acetone and isopropyl alcohol and ultrasonically cleaned for 30 min each. The cleaned ITO-glass substrates were exposed to a UV-ozone (UVO) environment for 20 min to remove some organic residues on the ITO surface. On the top part of the UVO-treated ITO-glass substrates, the P3HT solutions were spun and soft-baked at 60°C for 15 min. The thickness of the resulting P3HT layers was 50, 150, and 300 nm due to the different concentrations of P3HT solutions. The P3HT-coated samples were transferred into a vacuum chamber inside an argon-filled glovebox system. Then, the aluminum (Al, 95 nm) electrodes were deposited on the P3HT layers at a base pressure of ∼ 1.0 × 10^-6^ torr. Finally, the Al-deposited samples (devices) were thermally annealed at 140°C for 30 min.

#### Measurements

The thickness of P3HT layers and electrodes was measured using a surface profiler (DektakXT-E, Bruker). The cross-section of devices was measured using a scanning electron microscope (S-4800, Hitachi High-Technology, Japan). The optical absorption spectra of P3HT layers (coated on the ITO-glasses) were measured using a UV-visible spectrometer (Lambda 750, PerkinElmer, USA). The current density-applied voltage (J-V_APP_) curves of devices were measured using a home-built measurement system equipped with an electrometer (Keithley 2400, USA) and a semiconductor parameter analyzer (Keithley 4200 SCS, USA). The time-dependent current and voltage characteristics under pressing were measured using the same measurement system connected to a semiconductor parameter analyzer (Keithley 4200 SCS, USA). The pressure between the bottom (ITO-glass) and top (Al electrode) parts of devices was controlled via a double acting cylinder assembly controlled by a pressure controller (Model 2001, CHIYODA SEIKI, Japan). All devices were measured in the dark condition to avoid photovoltaic effects.
