# Supplementary material for: Persistent electrical energy generation from organic diodes under constant pressure: toward organic gravity nanogenerators
Source: iScience. 2021 May 15;24(6):102546. doi: 10.1016/j.isci.2021.102546 (PMC8184510; doi:10.1016/j.isci.2021.102546)
Supplement: Document S1. Figures S1–S5 and Table S1 [file mmc1.pdf]

iScience, Volume *24*

Supplemental information

Persistent electrical energy generation from organic diodes under constant pressure: toward organic gravity nanogenerators

Sooyong Lee, Hwajeong Kim, and Youngkyoo Kim

Supplemental Information

Table S1. Summary of device parameters (averaged for each day) during long-term pressing (7 days) for organic diodes with the 150 nm-thick P3HT layers in the dark and inert condition.

The constant pressure (5 kg/cm^2) was applied continuously on the devices.

**Parameters**	**Pressing Time (day)**						
	**1**	**2**	**3**	**4**	**5**	**6**	**7**
**V_P (μV)**	60.2	57.0	53.7	51.3	50.8	50.6	50.3
**I_P (μA)**	57.6	55.1	53.0	51.3	50.3	50.2	50.2
**P_P (nW)**	3.47	3.14	2.85	2.63	2.55	2.54	2.53

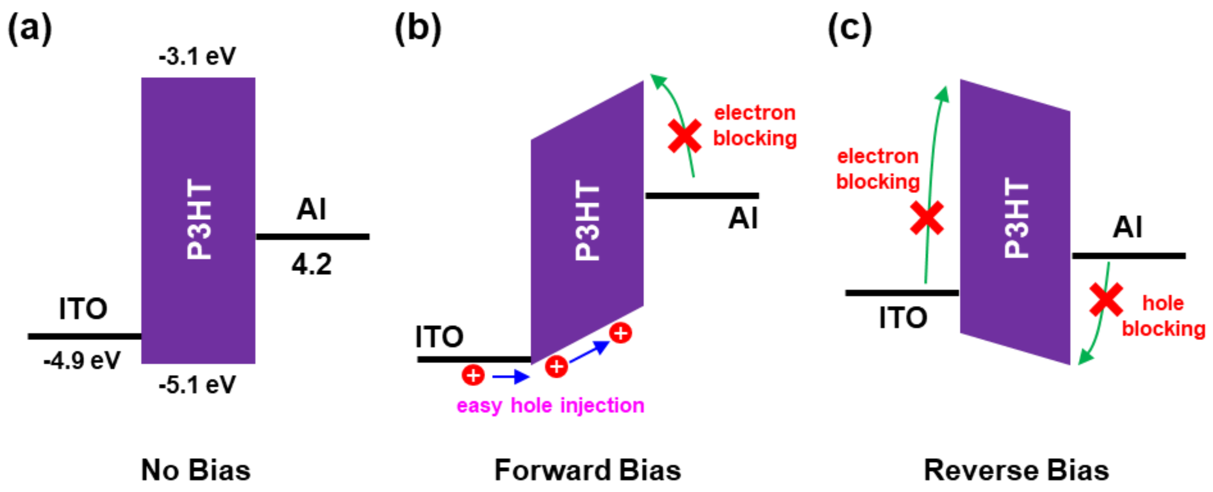

Figure S1. Brief illustration for the change of energy band diagram leading to the rectification behavior in the ITO/P3HT/Al diode devices.

(a) flat energy band diagram (a built-in electric field is not shown), (b) energy band diagram at forward bias condition, and (c) energy band diagram at reverse bias condition. In the case of forward bias, the hole charge carriers can be easily injected from the ITO electrode to the P3HT layer because of the small energy barrier between the ITO work function and the P3HT HOMO energy level (note that no electrons can be injected owing to the high energy barrier between the Al work function and the P3HT LUMO energy level). In the case of reverse bias, both holes and electrons cannot be injected into the P3HT layers because of the large energy barrier.

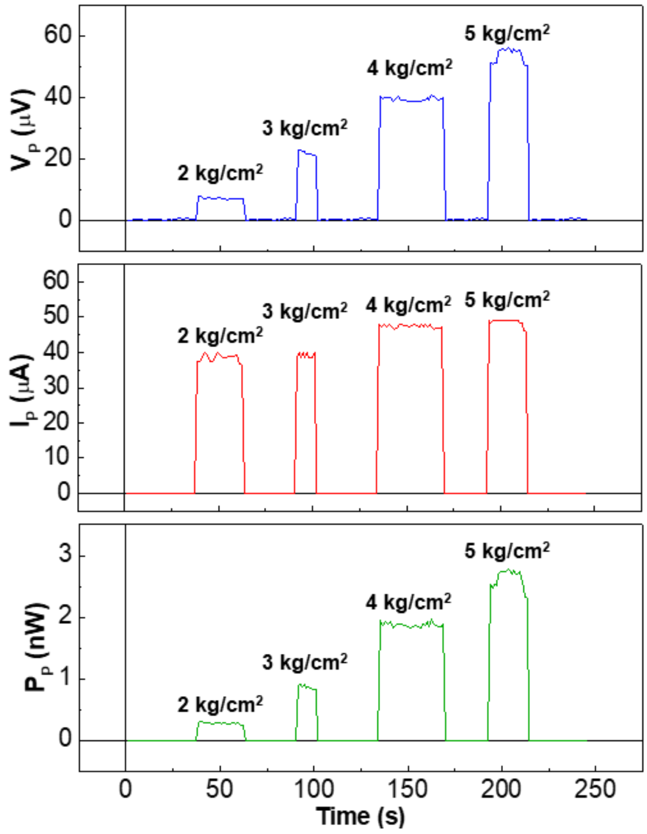

Figure S2. Short-term voltage and current signals generated from the organic diode devices with the 150 nm-thick P3HT layers by pressing according to the pressure change (2 ~ 5 kg/cm^2).

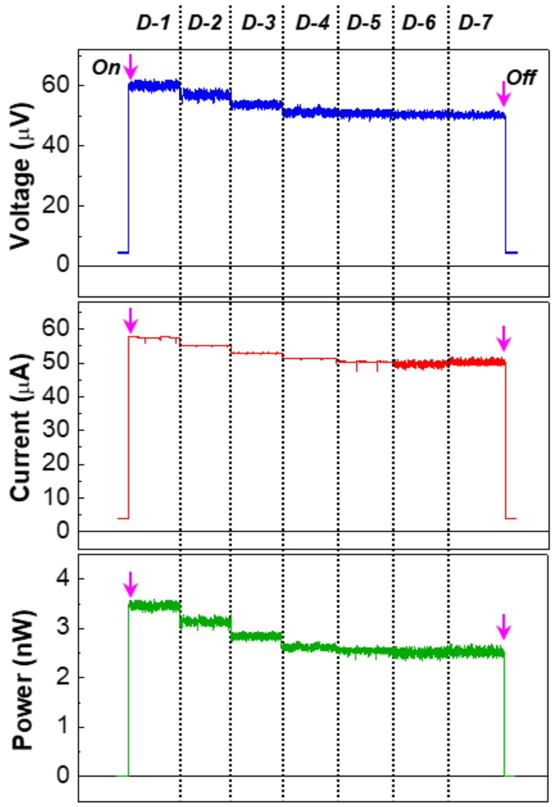

Figure S3. Long-term voltage and current signals generated from the organic diode devices with the 150 nm-thick P3HT layers upon continuous pressing (pressure = 5 kg/cm^2) for 7 days (D-1 ~ D-7). Note that the continuous data acquisition was run for one day (24 h) only, then the measurement system was turned off and turned on again for the next day measurement because of the limited memory chip spaces for data storage in the present measurement system. The arrows in pink indicate the starting and ending points of pressing.

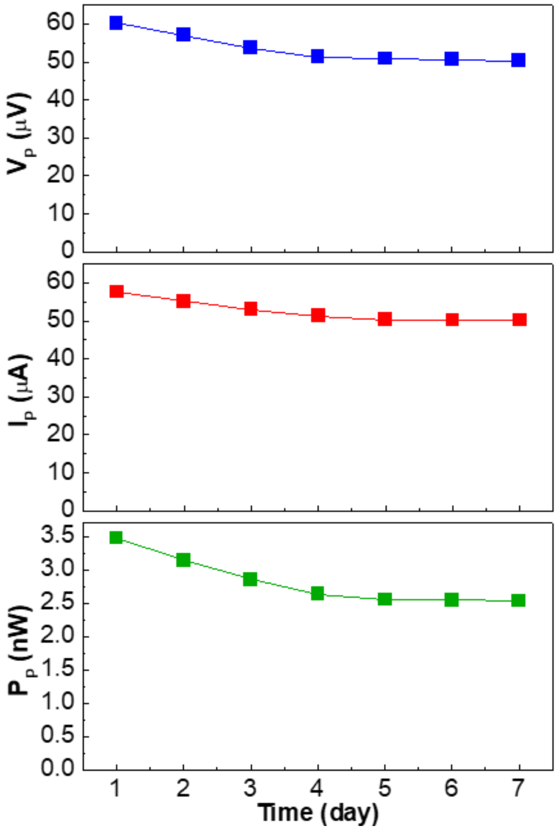

Figure S4. Long-term voltage and current signal values (averaged data for each day in Figure S3) as a function of pressing time (day).

Note that the average output voltage and current values were slightly decreased up to the third day (during three days) but the decreasing trend was almost stopped from the fourth day.

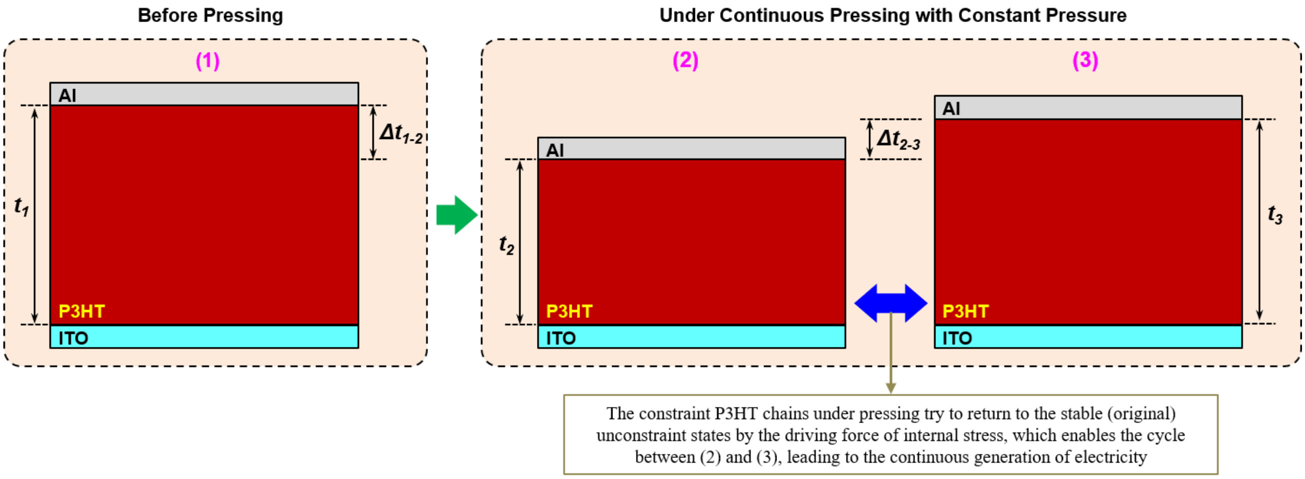

Figure S5. Illustration for a possible thickness change in the P3HT layers of organic diode devices under continuous pressing with constant pressure for long time.

Note that t_1, t_2, and t_3 represent the thickness of P3HT layers before pressing (1), under pressing but high constraint states leading to contraction of P3HT chains (2), and under pressing but low constraint states after expansion of P3HT chains by the internal stresses in the P3HT layers (3), respectively. The thickness difference ($\Delta t_{2\text{-}3}$) between (2) and (3) states is expected to be smaller than that ($\Delta t_{1\text{-}2}$) between (1) and (2) because of the pressed states (2 and 3).
